# Treatment of Multiple RT1 Gingival Recessions Using a Coronally Advanced Flap Associated with L-PRF or Subgingival Connective Tissue Graft from Maxillary Tuberosity: A Randomized, Controlled Clinical Trial

**DOI:** 10.3390/dj12040086

**Published:** 2024-03-28

**Authors:** Giuseppe Balice, Michele Paolantonio, Matteo Serroni, Paolo De Ninis, Imena Rexhepi, Alessio Frisone, Stefania Di Gregorio, Luigi Romano, Bruna Sinjari, Giovanna Murmura, Beatrice Femminella

**Affiliations:** 1 Department of Innovative Technologies in Medicine and Dentistry, University “G. d’Annunzio” Chieti-Pescara, Chieti, Italy; giuseppe.balice@phd.unich.it (G.B.); matteo.serroni@unich.it (M.S.); imena.rexhepi@unich.it (I.R.); alessio.frisone@unich.it (A.F.); stefania.digregorio@unich.it (S.D.G.); luigi.romano@unich.it (L.R.); b.sinjari@unich.it (B.S.); giovanna.murmura@unich.it (G.M.); beatrice.femminella@yahoo.it (B.F.); 2 “Luisa D’Annunzio” Institute for High Culture, Pescara, Italy; paolo.denins@conservatoriopescara.it

**Keywords:** periodontal plastic surgery, gingival recession, connective tissue graft, maxillary tuberosity, leukocyte and platelet-rich fibrin

## Abstract

The goal of plastic periodontal surgery is to obtain complete root coverage, increasing gingival thickness (GT), a positive prognostic factor for gingival margin stability over time. The aim of this study was to compare the effectiveness of the Coronally Advanced Flap (CAF) in RT1 (GR; gingival recession with no loss of interproximal attachment) when associated with a connective tissue graft from the maxillary tuberosity (tCTG) or with leukocyte and platelet-rich fibrin (L-PRF) membranes in obtaining root coverage and increasing the thickness and width of the keratinized tissue, along with aesthetic improvement, taking into account a number of patient-related outcomes. Thirty patients with two adjacent RT1 GRs (GRs with no loss of interproximal attachment) were each treated using CAF associated with tCTG (15 patients) or L-PRF. The main outcome was a GT increase; secondary outcomes were keratinized tissue width (KT), gingival recession (GR), probing depth (PD), clinical attachment level (CAL), root coverage percentage (RC%), complete root coverage (CRC), and root coverage esthetic score (RES). Patient-reported outcomes were discomfort (D), dentine hypersensitivity (DH), patient-related esthetic score (PRES), and overall treatment satisfaction (OTS). After 12 months, clinical and patient-reported parameters did not show significant differences between groups, with the only exception being a GT gain, which was significantly greater in the CAF + tCTG group. Our results showed that both techniques were effective in treating RT1 GRs, with comparable patient-related outcomes. However, the use of tCTG produces significantly thicker tissue, covering the exposed root surface.

## 1. Introduction

Gingival recession (GR) is the displacement of the gingival margin apical to the cemento-enamel junction (CEJ) [1], with a prevalence ranging from 40% to 100% in different populations and age groups [2]. In the presence of GRs, root surface exposure may be associated with dentine hypersensitivity, root caries, and esthetic discomfort [3,4,5]. Furthermore, untreated GRs tend to progress over time, even in highly motivated patients [6,7], necessitating surgical treatment.

Various techniques have been proposed for treating GRs; Zucchelli & Mounssifs classified surgical procedures as rotational flap procedures (lateral sliding flap, double papilla flap, oblique rotated flap); advanced flap procedures (coronally repositioned flap, semilunar coronally repositioned flap); free soft-tissue graft procedures (epithelialized graft, subepithelial connective tissue graft), or tunnel techniques [8]. Currently, the gold standard treatment for root coverage with long-term stability is connective tissue graft (CTG) plus a Coronally Advanced Flap (CAF), ensuring adequate gingival thickness [9]. Autogenous CTG acts as a biological scaffold and is considered the best material for treating gingival recessions [8]. It enhances flap stability over the root surface while increasing tissue thickness and keratinized tissue width [10,11,12]. The choice of the CTG harvesting site usually depends on individual clinician preferences, but it can also influence surgical outcomes [10]. Some authors have observed that connective tissue from the palatine sub-epithelial tissue, richer in dense connective tissue, is more suitable for root coverage, producing a greater increase in buccal soft tissue thickness [13].

CTG can induce the keratinization of the overlying epithelium, particularly if the graft is rich in collagen and lamina propria [14]. However, CTG from the deep palate may not induce keratinization as effectively as CTG from the superficial palate [15], possibly due to the presence of glandular and adipose tissue, which could impede vascularization and the plasmatic diffusion required for early healing [16].

Harvesting superficial connective tissue from the palate with the de-epithelialized gingival graft technique results in a wound that heals via secondary intention, causing pain and discomfort during chewing, especially in the early stages of healing. To obtain an optimal graft while reducing discomfort and morbidity, maxillary tuberosity has been proposed as a harvesting site for CTG. Sanz-Martin et al. [17] reported a higher percentage of collagen and lamina propria and a lower percentage of submucosa in the tuberosity area compared to a conventional palatal graft. Harvesting the graft from the tuberosity area also reduces the risk of intraoperative and postoperative problems and patient morbidity due to the reduced involvement of that anatomical area during chewing [10,18,19].

Dellavia et al. [15] compared the clinical and histological outcomes of tissue produced by CTG harvested from the palatal area and from the maxillary tuberosity area, observing a greater gain in soft tissue thickness and collagen cross-linking when tuberosity was used as the donor site.

Recently, García-Caballero et al. [20] confirmed that, from a histological standpoint, the best donor site is tuberosity because it consists solely of a thick lamina propria without the presence of a loose submucosal layer.

Asparuhova et al. [21] investigated the molecular profiles and associated functionalities of CTGs harvested from different locations and depths of the palate, concluding that they appear to be location-dependent (more favorable for root coverage in the posterior palate and tuberosity) rather than depth-dependent (superficial or deep palate).

Amin et al. [22] reported similar root coverage percentages when using grafts from the palate or the maxillary tuberosity; however, they found significantly less postoperative pain and discomfort when the graft was harvested from the maxillary tuberosity.

Another approach to reducing patient discomfort is the use of leukocyte- and platelet-rich fibrin membranes (L-PRF) as an alternative to CTG in the treatment of GRs [23], avoiding the need for a second surgical site for tissue harvesting.

L-PRF is a second-generation platelet concentrate with a three-dimensional fibrin network enclosing platelets and leukocytes capable of releasing growth factors and cytokines (e.g., TGF-ß, PDGF, VEGF, EGF, IGF-1, IL-1ß, IL-4, IL-6, TNF-α), promoting neo-angiogenesis, the proliferation of fibroblasts, and the migration of epithelial cells. These properties potentially increase the thickness and width of keratinized tissue (KT) [24,25]. It has been clinically demonstrated that, when L-PRF is associated with a CAF procedure, it does not enhance root coverage but increases gingival thickness (GT) at the grafted site [26].

Considering the biological properties of L-PRF and its ability to increase soft tissue thickness [27,28,29,30], we chose maxillary tuberosity as the CTG harvesting site due to its histological characteristics—the richness of the lamina propria and the scarcity of submucosa—to design a randomized comparative study with an increase in GT as the primary outcome. Furthermore, considering that the literature reports a low level of morbidity using both L-PRF grafts and tCTG, this parameter was also comparatively assessed, together with other clinical and patient-related outcomes, when treating multiple RT1 GRs ( lesions with no loss of interproximal attachment). Interproximal CEJ is not clinically detectable at either mesial or distal sites [31].

## 2. Material and Methods

### 2.1. Study Design and Population

**Figure 1:**
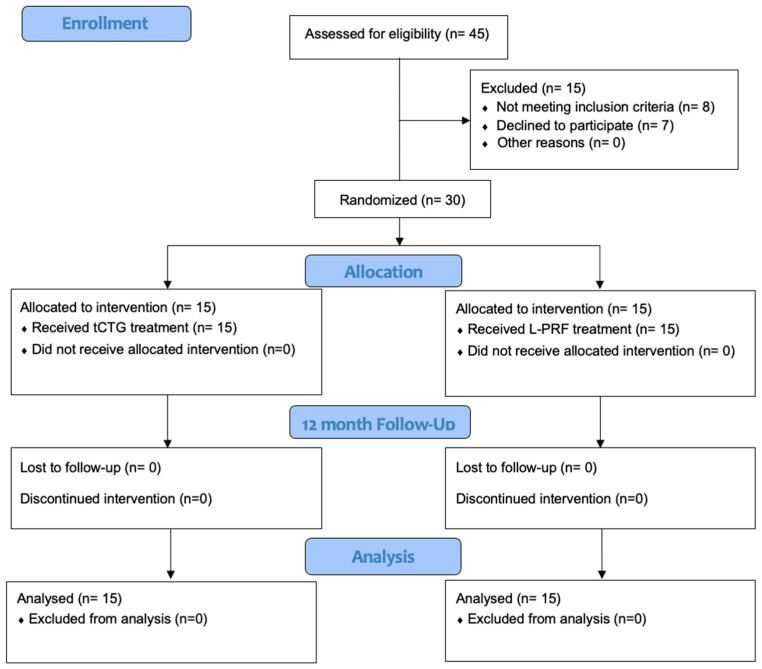
Study flow chart CONSORT diagram.

The study was designed as a parallel two-arm randomized and controlled clinical trial (the consort checklist is in the Supplementary Materials), conducted at the Unit of Periodontology and Dental Hygiene of the “G. D’Annunzio” University of Chieti–Pescara between October 2022 and November 2023 (Figure 1). The study protocol was approved by the “G. D’Annunzio” University Ethical Committee (n°1092–17 September 2021) following the principles of the Helsinki Declaration, as revised in 2013. Informed consent was obtained from all patients participating in the study.

This trial is registered at ClinicalTrials.gov as NCT05783258.

The first group was treated with CAF+t CTG, while the second group received CAF+L-PRF. Patients’ allocation to groups was randomized, with allocation concealed using opaque envelopes. Patients, examiners, and data analyzers were fully blinded; the surgeon was unaware only until the envelopes were opened. The inclusion criteria were: (1) age ≥ 18 years; (2) good systemic condition; (3) two adjacent RT1 GRs; (4) bilateral presence of adequate tissue distal to the second maxillary molars in the absence of third molars; (5) full mouth plaque score (FMPS) and full mouth bleeding score (FMBS) <20% at baseline; (6) absence of subgingival restorations at the sites to be treated; (7) non-smoker; (8) absence of pregnancy/lactation; (9) absence of periodontitis.

The primary outcome of the study was GT gain. Secondary outcomes included a KT increase, probing depth (PD) change, clinical attachment level (CAL) gain, GR reduction, complete root coverage (CRC) fraction, root coverage percentage (RC%), root coverage esthetic score (RES) [11], postoperative discomfort (D), reduction in dentinal hypersensitivity (DH), patient-related esthetic score (PRES), and overall treatment satisfaction (OTS).

### 2.2. Sample Size and Randomization

The sample size was calculated to detect an average group difference of 0.7 mm in the main outcome, assuming a standard deviation (SD) of 0.5, as reported by Amin et al. [22]. Fourteen patients per group were initially required, which was increased to fifteen.

Patients were randomly assigned to the experimental groups using a custom-made, computer-generated table.

### 2.3. Clinical Measurements

All clinical parameters were measured using a UNC-15 periodontal probe by the same clinician (MS), who underwent previous intra-examiner calibration exercises. This calibration exercise involved measuring GT in 20 patients twice, 24 h apart, to ensure a weighted Cohen’s Kappa value of ≥0.6. Measurements were taken at baseline (t_0_) and 12 months after surgery (t_1_). The clinical parameters analyzed included: probing depth (PD), evaluated as the distance between the gingival margin and the bottom of the sulcus; clinical attachment level (CAL), measured as the distance between the cemento-enamel junction (CEJ) and the bottom of the sulcus; keratinized tissue (KT), measured as the distance from the mid-buccal site of the gingival margin to the mucogingival junction; gingival recession (GR), measured from the CEJ to the gingival margin; complete root coverage (CRC), expressed as the percentage of completely covered GRs; root coverage percentage (RC%), representing the percentage of exposed root covered after treatment.

Gingival thickness (GT) was measured using a “bone sounding” technique with a k-file #15 inserted perpendicular to the gingiva until bone contact was made. A disk stop was placed in contact with the gingiva and fixed with cyanoacrylate. GT was determined as the distance from the reamer’s tip to the silicon disk and measured with a digital caliper accurate to 0.1 mm (Newaner^®^ via Milano 43, Prato, Italy).

### 2.4. Patient-Reported Outcomes

Two weeks after surgery, at suture removal, the patients’ postoperative morbidity (D) was evaluated using a visual analog scale (VAS) score. Dentine hypersensitivity was recorded at t_0_ and t_1_ using the Schiff cold air sensitivity scale [32]. The examiner, after protecting adjacent teeth, directed a jet of air at 60 psi from a dental syringe, 10 mm from the cervical area of the teeth. The patient’s response was then quantified on a 0–3 scale (0: no reaction to air stimulus, 1: reaction without requiring termination, 2: reaction with a request to stop, 3: painful stimulus complaint). At the 12-month follow-up, the Patient-Related Esthetic Score (PRES) was assessed on a VAS scale (0–10), indicating the level of esthetic satisfaction. This assessment involved comparing two standardized photos: the first taken at t_0_ and the second taken at t_1_. Furthermore, patients’ OTS was evaluated at t_1_ by asking whether they would undergo surgery again (yes/no), considering the outcome and the pain experienced.

### 2.5. Esthetic Outcome

The esthetic result for each recession was evaluated at the 12-month follow-up using the root coverage esthetic score (RES) [11].

### 2.6. Clinical Procedure

#### 2.6.1. Pre-Surgical Treatment

After patient inclusion, oral hygiene instructions, ultrasonic instrumentation, and supragingival polishing were performed. Furthermore, patients were informed about factors favorable to the progression/development of recession, and the use of an electric toothbrush with controlled pressure and extra-soft bristles (Oral-B Professional Care 1000, made in Germany) was recommended. After 2 weeks, patients were recalled for clinical measurements at t_0_.

#### 2.6.2. tCTG Preparation

**Figure 2:**
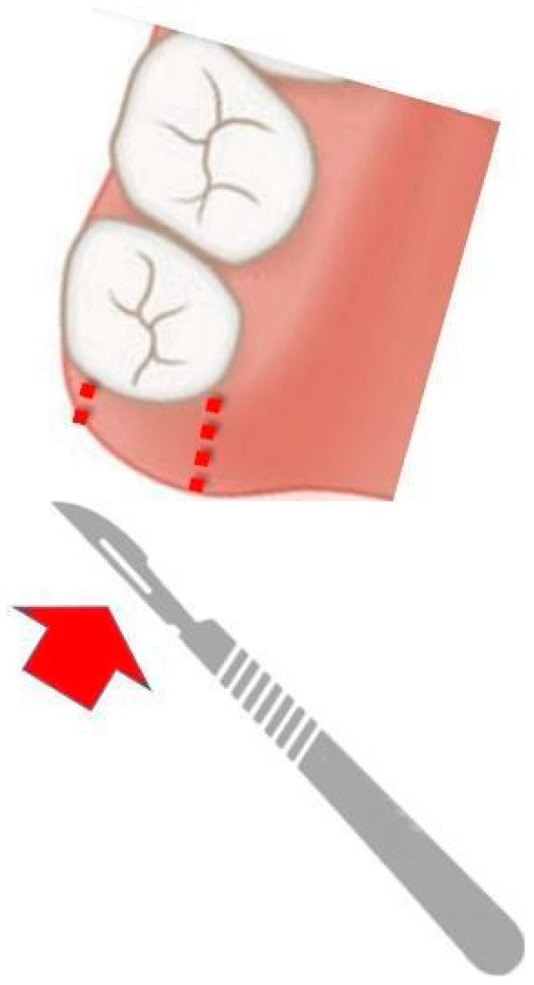
Distal gingivectomy for tCTG harvesting. The arrow indicates the cutting direction and is intuitive.

In the group treated with tCTG, the donor sites were identified in the distal area to the second maxillary molars. After local anesthesia, two CTGs (one for each recession in each patient) were bilaterally harvested by performing distal gingivectomy (Figure 2). The 15c scalpel blade was kept perpendicular to the retromolar mucosal planes to delineate a clean cut from distal to mesial until reaching the distal surface of the second maxillary molar.

**Figure 3:**
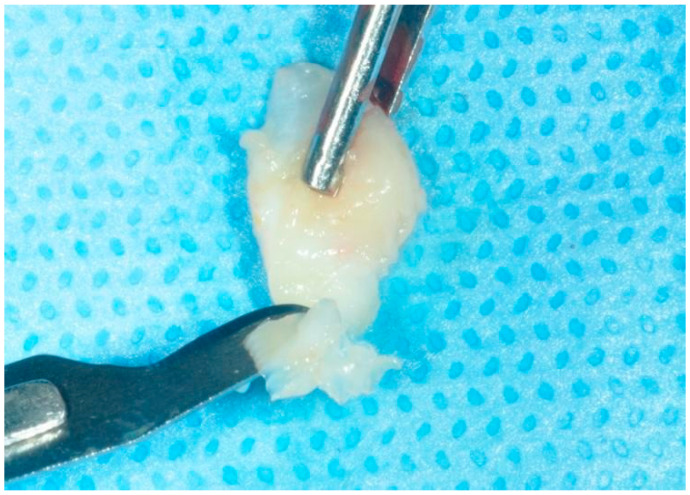
tCTG de-epithelialization.

**Figure 4:**
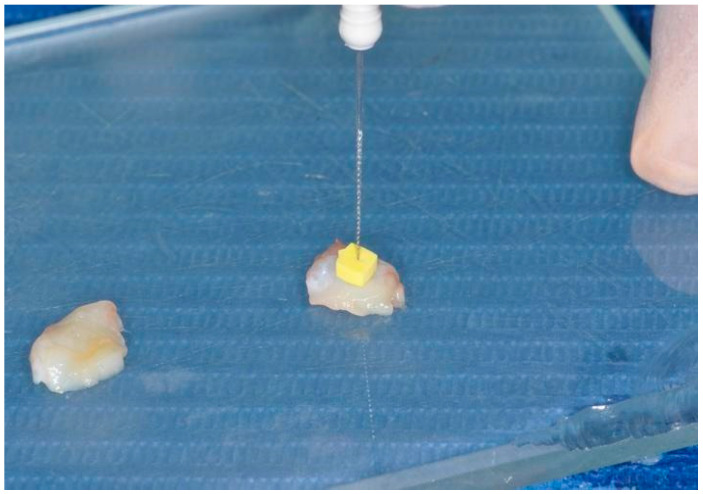
Tuberosity graft measurement using sterile k-file #15.

Subsequently, the grafts were de-epithelialized by removing the thin epithelial layer using a scalpel blade (Figure 3). The thickness of the grafts was standardized using a digital caliper and a k-file #15 to obtain an approximately 2 mm thick graft (Figure 4).

#### 2.6.3. L-PRF Preparation

**Figure 5:**
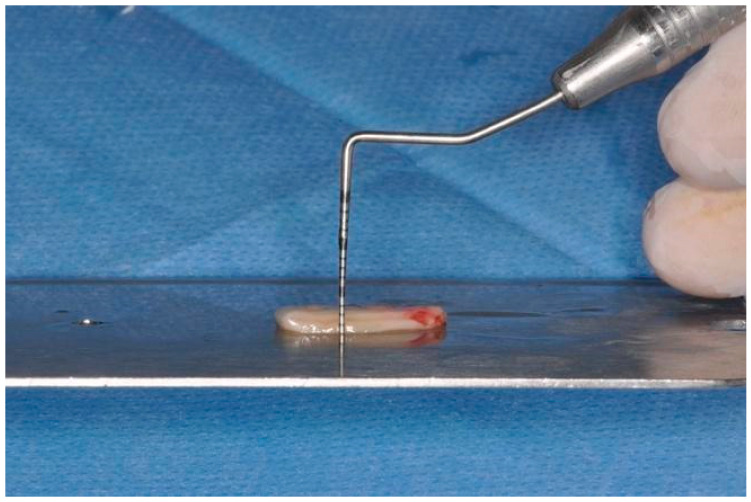
Two membranes were superimposed to create a double layer of L-PRF of about 2 mm thickness.

In the group treated with platelet concentrate, L-PRF was prepared according to the protocol by Choukroun et al. [33]. Immediately before surgery, a venous blood sample was collected from the antecubital vein into two 10 mL sterile tubes without anticoagulant. Subsequently, the blood sample was centrifuged (IntraSpin™, Intra-Lock System Europa SpA, Salerno, Italy) at 3000 rpm for 10 min. The fibrin clot was collected and squeezed into the L-PRF Box (Xpression™ Fabrication Kit, Intra-Lock System Europa SpA, Salerno, Italy) to obtain two L-PRF membranes. For each patient, the membranes were superimposed to create a double layer approximately 2 mm in thickness (Figure 5).

#### 2.6.4. CAF Procedures

**Figure 6:**
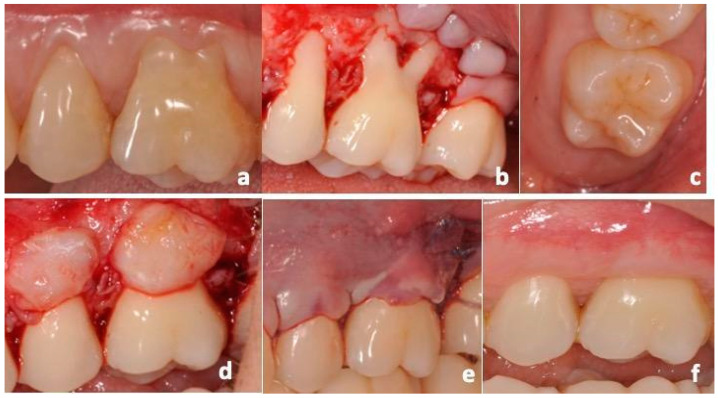
tCTG group surgical procedures: (**a**) RT1 GRs affecting 25 and 26 elements; (**b**) flap design and elevation; (**c**) maxillary tuberosity harvesting area; (**d**) grafts stabilization; (**e**) coronal repositioning of the flap and suturing; (**f**) 12-month follow-up.

**Figure 7:**
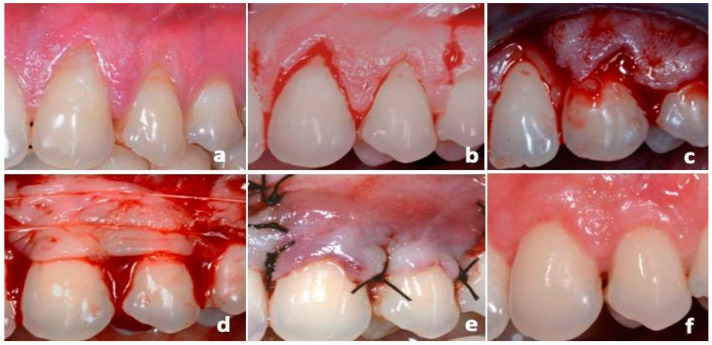
L-PRF group surgical procedures: (**a**) RT1 GRs affecting 23 and 24 elements; (**b**) flap design; (**c**) tension-free flap; (**d**) positioning and suturing of L-PRF-membranes; (**e**) coronal repositioning of the flap on the surgical papillae; (**f**) 12-month follow-up.

All patients were treated by the same experienced surgeon (MP). Local anesthesia was administered using mepivacaine 2% with 1:100,000 epinephrine (Mepivacaina Pierrel Pharma srl, Capua, Italy), and the exposed root surfaces were scaled using Gracey curettes. Coronally Advanced Flap (CAF) was performed according to the Allen and Miller technique [34]: a tension-free trapezoidal flap was designed, and two vertical incisions were made, beginning at the mesial line-angle of the first recession and at the distal line-angle of the distal gingival recession (GR), at a distance from the tip of each papilla equal to the dimension of the recession and extended obliquely beyond the mucogingival junction. Intrasulcular incisions were made around the teeth with recession defects, enclosing the interdental papilla. A split-thickness flap was raised, and the papillae mesial and distal to the surgical flap were de-epithelialized to produce an enlarged recipient bed. The flap was then placed coronally, remaining in place passively. Citric acid conditioning was not performed. The tCTG and the double-layer L-PRF membranes were positioned on the exposed roots, below the cemento-enamel junction (CEJ), and were stabilized with 3-0 PGA compressive sutures (Hu-Friedy, Milan, Italy). Then, the flap was coronally positioned and secured with interrupted sutures (Figure 6 and Figure 7).

### 2.7. Postoperative Care

Amoxicillin and clavulanic acid (1 g/12 h) were employed for 6 days, and ketoprofen 80 mg was to be used if necessary; two daily chlorhexidine digluconate 0.2% mouthwashes (Dentosan 0.20, Johnson & Johnson, Pomezia, Italy) were prescribed. All patients were instructed not to brush or traumatize the post-surgical area and to apply chlorhexidine gel (Corsodyl gel, GlaxoSmithKline Consumer Healthcare S.p.A.-Baranzate, Italy) locally, four times a day, for one month after surgery. Subjects were recalled monthly in the first 6 months after surgery and subsequently every 3–6 months for oral prophylaxis. At 12 months after surgery (t_1_), the patients underwent final evaluation (Figure 6f and Figure 7f).

## 3. Statistical Analysis

CRC fraction along with OTS were analyzed by means of Fisher’s exact test and logistic regression. The outcomes in VAS-Scale with DH, RES scores, and PRES were analyzed using cumulative logistic models, reporting the latent-variable results provided by the R package emmeans with mode = “mean.class” (means of the ordinal response, to be interpreted as a numeric value from 1 to the number of classes). All remaining parameters were analyzed using linear fixed-effect models. Since each patient contributed two recession sites, the statistical unit was expressed as the average value for each patient (No-pooling, highest-level means analysis). The root cover percentage model was interactive and was probed by means of the Johnson–Neyman technique. CAL gains and GR reductions were analyzed by means of ANCOVAs with CAL_t0_ and GR_t0_ as covariates; GT gain and KT gain, on the other hand, were analyzed by means of ANOVA models.

## 4. Results

### 4.1. Study Population

All 30 patients (17 women and 13 men, aged 18–67 years) completed the 12-month follow-up without any postoperative complications.

The tests conducted at baseline between groups, although not providing information about group comparability at baseline, were reported as a check of the randomization efficacy (see Table 1). Throughout the investigation, there were no group differences observed in FMBS or FMPS, which remained <20%.

### 4.2. Primary Outcome, Clinical Treatment Outcomes

**Figure 8:**
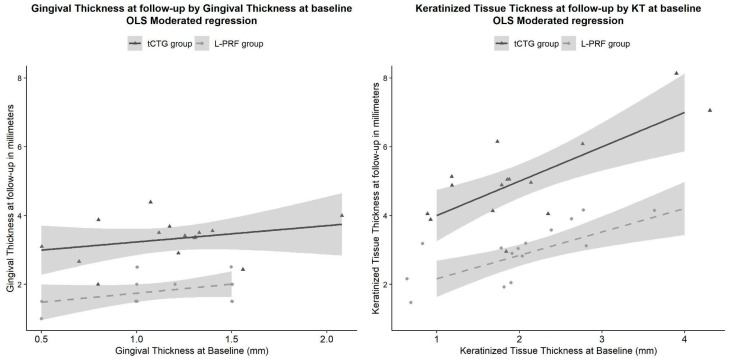
Gingival thickness and keratinized tissue by respective baseline values in the treatments: although the selected models for these parameters were ANOVAs, the moderated models are shown here to allow the reader an unconstrained appreciation of the treatment’s effect and of the sustainability of the assumption of homogeneity of the regression slopes needed for the ANCOVA analysis.

At the 12-month evaluation, both treatments resulted in an improvement of clinical outcomes from baseline. GT improved in both groups, with tCTG showing a mean improvement of 2.14 mm [1.79 to 2.49] and L-PRF showing a mean improvement of 0.69 mm [0.44 to 0.94]. The GT gain with tCTG was 1.45 mm [1.05 to 1.86] more than that of L-PRF (*p* < 0.001) (Figure 6, left). Additionally, a keratinized tissue (KT) gain occurred in both groups, with tCGT showing a mean gain of 3.00 mm [2.54 to 3.47] and L-PRF showing a mean gain of 0.8 mm [0.47 to 1.13]. However, the tCTG gain was 2.2 mm [1.65 to 2.75] larger than L-PRF (Figure 8, right).

Both techniques had proven equally effective in GR reduction, tCTG = 3.13 [2.55 to 3.72] mm L-PRF = 3.00 [2.64 to 3.36], with inconclusive differences between group tCTG − L-PRF = 0.133 [−0.218 to 0.484] mm. The RC% model included the treatment predictor, the GR_t0_ covariate, and treatment using GR_t0_ interaction:

RCP = β_0_ + β_1_ · GR_t0_ + β_2_ · treatment + β_3_·GR_t0_ · treatment + ε

whose estimated parameters and R^2^ are the following:
***Predictors*****Dependent Variable*****Estimates******CI******p***(Intercept)117.7385.66–149.81<0.001GR_t0_−8.45−17.41–0.520.064TREAT [tCTG]−46.91−90.69–−3.130.037GR_t0_ × TREAT [tCTG]13.691.48–25.900.029Observations30R^2^/R^2^ adjusted0.173/0.078

**Figure 9:**
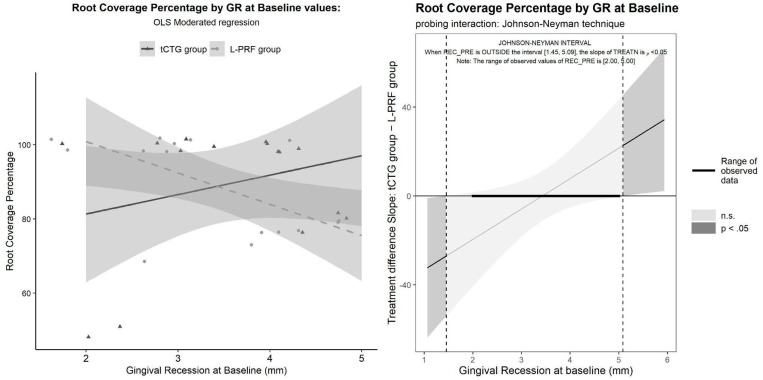
Coverage percentage by GR baseline by treatment: OLS-moderated model with probing using the Johnson–Neyman technique.

The interaction term being significant (*p* = 0.029), it was subjected to probing using the Johnson–Neyman technique. The significance region of the difference between treatments was relating to GR_t0_ values outside the interval 1.45 to 5.09. The interaction was disordinal: for GR_t0_ < 1.45, tCTG was significantly inferior to L-PRF; meanwhile, for GR_t0_ > 5.09, the opposite was true (see Table 2 and Figure 9).

**Figure 10:**
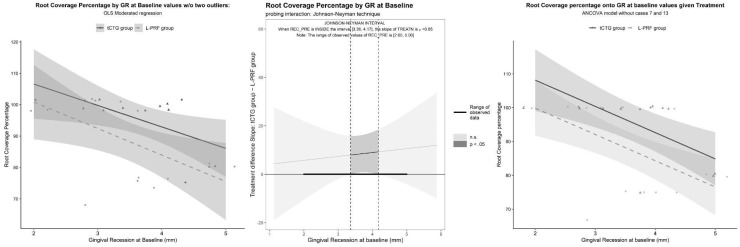
Coverage percentage by GR baseline by treatment removing cases 7 and 13: OLS-moderated model, probing with the Johnson–Neyman technique, and ANCOVA final model.

Given that the GR_t0_ range of the sample (2 to 5 mm) is entirely within the significance region, the result is inconclusive. However, the implausibility of the worse performance of tCTG at a small GR_t0_, along with two observations (bottom-left in the plot) being suspect outliers, prompted us to conduct a sensitivity analysis by removing them. The result of this analysis completely reverses the previous, suggesting the superiority of tCTG for all GR_t0_ ranges. After dropping the interaction term, which was nonsignificant (*p* = 0.71), an ANCOVA model was produced (see Figure 10), with the results reported in Table 2.

For PD, changes smaller than 1 mm were found from baseline to 12 months in both groups, with tCTG showing a change of 0.53 mm [−1.04 to −0.03] and L-PRF showing a change of 0.13 mm [−0.06 to 0.33] (Table 1). Although statistically significant, the difference between tCTG and L-PRF, −0.667 mm [−1.19 to −0.148], was not clinically relevant.

The difference in the complete root coverage (CRC) fraction obtained using the treatments was inconclusive. CRC was observed in 67% of patients treated with tCTG and 53% treated using L-PRF (Table 2).

### 4.3. Patient-Reported Outcomes

**Figure 11:**
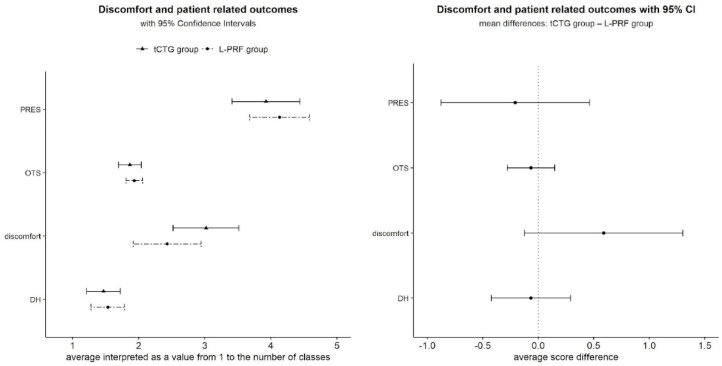
Patient-related outcomes, reported according to the *emmeans* R package with mode = “mean.class”.

No clinically relevant differences were found between groups for D (D = 0.588 ± 0.364, *p* = 0.11) and for DH (DH = −0.067 ± 0.182, *p* = 0.71). Almost all patients expressed satisfaction with the treatment received, with no clinical difference between the two surgical techniques (PRES = 0.41 ± 0.677, *p* = 0.54). The OTS difference was assessed at 0.067, OR = 0.464 ± 0.596, *p* = 0.55 (Figure 11).

### 4.4. Esthetic Outcomes

**Figure 12:**
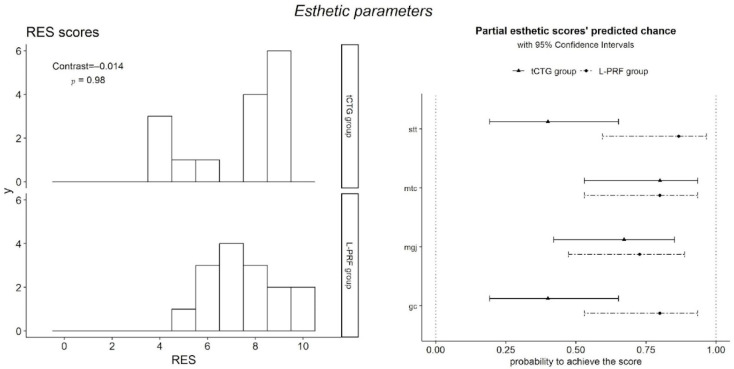
Esthetic outcomes: RES scores and their constituents.

Finally, no RES difference was assessed between the two groups (0.014 ± 0.638, *p* = 0.98). In the tCTG group, the arithmetic mean was 8 (5.5 to 9), while, in the group treated with L-PRF, it was 7 (6.5 to 8.5) (Table 2, Figure 12).

## 5. Discussion

This randomized clinical trial was designed to evaluate the clinical differences between two surgical techniques, CAF + tCTG and CAF + L-PRF, in the treatment of multiple RT1 GRs. Both techniques improved the clinical conditions similarly; however, GT showed significantly greater improvement in patients from the CAF + tCTG group. The greater capability of tCTG to produce a clinically significant gain in GT is explained by its specific histological and molecular features [15,17,35]. A histomorphometric study by Sthur et al. [35] showed a 1.5–2 mm thicker lamina propria in tCTG than in conventional CTG and a five-fold increase in COL1A1 gene expression in grafts from the maxillary tuberosity, producing denser, thicker tissue. The high amount of collagen, the over-expression of lysyl hydroxylase (LH), predominantly LH2b [15,17,35], and greater cross-linking between collagen molecules result in a denser tissue response [15,21,35]. However, it must be considered that although the greater GT provided using tCTG is considered a positive outcome, being a predictor of gingival margin stability over time [36,37,38,39], a propensity for fibrotic response may lead to hyperplastic outcomes in some patients and, therefore, to unsatisfactory aesthetic results [15]. The GT increase obtained from baseline to 12 months in patients treated with CAF + L-PRF, although statistically significant, was smaller than that obtained in the tCTG group.

We agree with the hypothesis that the better results achieved using CTGs are mostly due to their ability to stabilize the surgical flap and to improve the maturation of the blood clot, thus favoring an increase in GT [24,25,26,27,28,29,30]. On the contrary, the L-PRF membranes have a rapid resorption time, approximately 7–10 days, that is associated with reduced release times for both glycoproteins and growth factors [33] and a poor ability to stabilize the clot in the crucial initial phase of healing [33]; this explains the comparably reduced ability of L-PRF to promote an increase in GT. In a recent review, Mancini et al. [26] concluded that CAF + L-PRF is able to offer better results in terms of GT gain compared to CAF alone, but lower outcomes compared with CAF + CTG, which is in accordance with our results.

To the best of our knowledge, to date, there are no studies in the literature comparing the effectiveness of tCTG with L-PRF grafts at increasing gingival thickness. However, studies exist that separately evaluate the use of tCTG or L-PRF in association with CAF [15,22,24,25,31,32,33,40,41]. In agreement with our results, Amin et al. [22] obtained a GT gain of about 2.9 ± 0.5 mm in tCTG-treated GRs. Dellavia et al. [15], however, using tCTG, showed an increase in GT greater than ours, namely, of about 4.7 ± 0.9 mm, at 12 months post-surgery. It is necessary to note that Dellavia’s data come from a study conducted on ridge augmentation procedures and, therefore, are not directly comparable to our results.

In our study, tCTGs provided a greater GT gain than that achieved by patients treated with a traditional, lateral palatal harvest. Already in 2002, Paolantonio [42] evaluated a GT gain of ≥1.22 mm after a CAF + CTG; Zucchelli et al. observed a similar increase (1.55 ± 0.21 mm) [43]. Conversely, in a recent study, Santamaria et al. [44] reported a lower GT increase of 0.99 ± 0.02 mm using CAF+CTG. The GT gain shown by the L-PRF group is in line with what is described in the literature [31,33]. Aroca et al. [24], in a study with a follow-up of 6 months, showed a lower increase in GT gain (0.3 mm); instead, Santamaria et al. [44] estimated a GT increase of 0.92 ± 0.52 mm in CAF + L-PRF treated patients with no statistically significant difference compared with patients treated using CTG.

We hypothesize that the heterogeneity of the results on the increase in GT with the use of L-PRF may be associated with the different thicknesses of the L-PRF layer positioned on the exposed roots by different authors. In this regard, for example, Aroca et al. [24] placed a single L-PRF membrane on the exposed roots, while Santamaria et al. [44] used two membranes placed one over the other to obtain a 1.5 mm-thick L-PRF graft. In the literature, there is no uniform protocol for the creation of L-PRF grafts to be used in association with a CAF technique, and the amount of biological mediators contained in L-PRF may change from patient to patient [26,27,28,29,30,31,45].

The two surgical techniques compared in this study demonstrated similar effects in covering exposed roots. Both the variations in GR and CAL, as well as the RC% and CRC fraction, did not show significant differences between the experimental groups. These findings are in alignment with the literature that compares conventionally harvested CTG with L-PRF grafts [26,44].

From baseline to 12 months postoperatively, a statistically significant increase in KT width was observed in both groups. In the CAF + tCTG group, we hypothesized that the increase in keratinized tissue width was induced by the significant amount of lamina propria in the tCTG, associated with a notable quantity of cytokeratins [35,38].

The KT gain obtained in the CAF + PRF group was consistent with the current literature. Studies by Aroca et al. [24] and Kuka et al. [41] demonstrated an additional gain in KT width compared to CAF alone when using CAF + PRF. A recent systematic review by Mancini et al. [26] confirmed that CAF + PRF can produce a greater gain in KT width compared to CAF alone but lower than that obtained using CAF + CTG.

In our study, no unsatisfactory esthetic results occurred, both from an objective (RES) and subjective (PRES) standpoint, with no differences between groups. However, while optimal aesthetic results were expected in patients treated with CAF + L-PRF, as confirmed by the literature [24,41], CAF + tCTG was sometimes associated with an unpleasant aesthetic outcome due to the continuous hyperplastic tendency induced using tCTG, possibly related to the excessive thickness of the graft with molecular implications [15,21,35]. The hyperplastic tendency of tCTG has been reported mainly in case reports in periodontal plastic surgery [18] and in studies on ridge augmentations [15] and dental implants [46,47,48]. When hyperplasia was reported in root coverage procedures, it was observed at follow-ups longer than 12 months, exceeding the follow-up duration in our study. A controlled study by Amin et al. [22] with an eight-week follow-up reported no hyperplastic phenomena, similar to our findings. It is possible to hypothesize that the good aesthetic result appreciated by patients and clinically assessed through the RES may depend on the limited follow-up period and will need to be confirmed by studies with longer follow-up periods. Another possible explanation for the absence of hyperplastic phenomena can be attributed to the fact that, in our study, we used tCTGs no thicker than 2 mm, as suggested by Dellavia et al. [15].

All patients reported optimal PRES and OTS results, with no differences between groups. In particular, comparable OTS scores from both groups indicate that although tCTG harvesting requires a second surgical site, while the L-PRF graft only needs venipuncture, the graft from the maxillary tuberosity is comparable to the L-PRF graft in terms of patient morbidity, with a similar level of satisfaction.

This consideration may be taken into account when proposing a procedure characterized by a low level of discomfort despite requiring a significant increase in GT. This may be attributed to the greater thickness of the connective tissue left covering the underlying bone. Furthermore, the area of the tuberosity is less exposed to chewing friction and mechanical tongue stimulation [30].

Several studies in the literature [26,41,49] confirmed less postoperative discomfort and faster wound healing when using L-PRF membranes. L-PRF has also been proposed as a palatal bandage after traditional tissue harvesting from the palate [49].

The reduction in DH, similar in both groups, can be explained by the similar amount of root coverage obtained by the two techniques.

Our results may suggest that clinicians should use tCTG to treat GRs in patients with very thin gingival tissues, for whom a significant increase in GT is particularly desirable. However, the data reported in Table 2 and Figure 8 show that, although overall GR reduction does not differ significantly between the two experimental groups, for GRt_0_ > 5.09, the use of tCTG associated with CAF has a significant advantage over the use of L-PRF, making it the more suitable technique when the clinician has to treat very deep GRs.

Furthermore, tissue harvesting from the maxillary tuberosity with the gingivectomy technique is an extremely simple procedure for the clinician and makes the surgical procedure very quick.

However, it is worth noting that the use of the maxillary tuberosity as a donor site is not always feasible, depending on the patient’s “anatomical availability”.

Several limitations in our study can be highlighted.

The trial was conducted on multiple recessions (two adjacent recessions for each patient); thus, it remains to be seen whether similar conclusions can be confirmed when dealing with single recessions.

Furthermore, the experimental protocol does not follow a “split-mouth” design, which would have allowed for the removal of individual variability and provided more precise estimates along with greater power.

Moreover, the detection of clinical parameters was not assisted by the use of stents, which would have improved the repeatability of probing.

Gingival phenotype is a known prognostic factor for root coverage and GR recurrence [39]. It has been suggested that the use of a graft in addition to the CAF is indicated when the gingival thickness in the GR area is less than 0.8 mm [50]. In our study, only six patients (four in the tCTG group and two in the L-PRF group) had GTt_0_ smaller than the Cairo threshold (0.8 mm) [50,51]. Even though we observed (see Figure 6) that all achieved a minimal GTt_1_ of 1 mm, which increased to about 2 mm for the tCTG group, it would be helpful to determine whether this result could be confirmed by a whole sample of thin gingival phenotype patients, for whom these treatments have a specific indication.

Finally, the surgical technique we used did not adhere to the principles of minimally invasive periodontal plastic surgery, and this may have negatively influenced the esthetic results [52].

Other studies will be necessary to evaluate the potential hyperplastic response induced using tCTG, with a follow-up exceeding 12 months.

## 6. Conclusions

This is the first study to compare the effectiveness of tCTG with that of L-PRF associated with CAF. Both surgical techniques demonstrated usefulness in covering exposed roots in cases of RT1 GRs. Our results showed good clinical effectiveness of both surgical techniques with comparable patient-related outcomes. The tCTG group obtained a greater GT increase, suggesting that this technique may be indicated in cases where there is a particular need to increase gingival thickness. Other studies are required to confirm our results.

## Figures and Tables

**Table 1:** Clinical parameters observed means ± SD in millimeters (continuous variables), medians (discrete variables), or frequencies (categorical variables) n = 15 in each group. Tests of equality at baseline are shown because they are customary and only for descriptive purposes.

Parameter	Treatment	Baseline Mean ± SD (95% CI)	12 Months Mean ± SD (95% CI)	Baseline-12 Months Mean (95% CI)	Within-Group Differences
**GENDER** **(Male)**	CAF + tCTG	5 (33%)			
	CAF + L-PRF	6 (40%)			
	Between Groups Difference	NS			
**AGE**	CAF + tCTG	43.67 ± 16.12 (34.74 to 52.59)			
	CAF + L-PRF	43.27 ± 15.63 (34.61 to 51.92)			
	Between Groups Difference	NS			
**PD**	CAF + tCTG	1.27 ± 0.46 (1.01 to 1.52)	1.8 ± 1.01 (1.24 to 2.36)	−0.53 ± 0.92 (−1.04 to -0.03)	0.04
	CAF + L-PRF	1.2 ± 0.41 (0.97 to 1.43)	1.07 ± 0.26 (0.92 to 1.21)	0.13 ± 0.35 (−0.06 to 0.33)	NS
	Between Groups Difference	NS	*p* = 0.011	*p* = 0.013	
**CAL**	CAF + tCTG	4.73 ± 0.88 (4.24 to 5.22)	2.13 ± 0.99 (1.58 to 2.68)	2.6 ± 1.4 (1.82 to 3.38)	<0.001
	CAF + L-PRF	4.67 ± 0.9 (4.17 to 5.16)	1.53 ± 0.52 (1.25 to 1.82)	3.13 ± 0.64 (2.78 to 3.49)	<0.001
	Between Groups Difference	NS	*p* = 0.047	*p* = NS	
**GR**	CAF + tCTG	3.47 ± 0.99 (2.92 to 4.02)	0.33 ± 0.49 (0.06 to 0.6)	3.13 ± 1.06 (2.55 to 3.72)	<0.001
	CAF + L-PRF	3.47 ± 0.92 (2.96 to 3.97)	0.47 ± 0.52 (0.18 to 0.75)	3 ± 0.65 (2.64 to 3.36)	<0.001
	Between Groups Difference	NS	NS	NS	
**KT**	CAF + tCTG	2.07 ± 0.96 (1.53 to 2.6)	5.07 ± 1.28 (4.36 to 5.78)	3 ± 0.85 (2.53 to 3.47)	<0.001
	CAF + L-PRF	2.13 ± 0.83 (1.67 to 2.6)	2.93 ± 0.78 (2.5 to 3.36)	0.8 ± 0.59 (0.47 to 1.13)	<0.001
	Between Groups Difference	NS	*p* < 0.001	*p* < 0.001	
**GT**	CAF + tCTG	1.17 ± 0.38 (0.96 to 1.39)	3.31 ± 0.62 (2.97 to 3.66)	2.14 ± 0.63 (1.79 to 2.49)	<0.001
	CAF + L-PRF	1.11 ± 0.34 (0.93 to 1.3)	1.8 ± 0.46 (1.55 to 2.05)	0.69 ± 0.45 (0.44 to 0.94)	<0.001
	Between Groups Difference	NS	*p* < 0.001	*p* < 0.001	
**Patients obtaining** **Complete Root Coverage**	CAF + tCTG		10 (67%)		
	CAF + L-PRF Between Groups Difference		8 (53%) NS		
**Root Coverage %**	CAF + tCTG		89 ± 18.15 (78.95 to 99.05)		
	CAF+ L-PRF		88.45 ± 13.11 (81.19 to 95.71)		
	Between Groups Difference		NS		
**RES score**	CAF + tCTG		8 (5.5 to 9)		
	CAF + L-PRF		7 (6.5 to 8.5)		
	Between Groups Difference		NS		
**Pain (VAS)**	CAF + tCTG		4 (3 to 5)		
	CAF + L-PRF		4 (2.5 to 4)		
	Between Groups Difference		NS		
**Dentine Hypersensitivity**	CAF + tCTG	2 (2 to 3)	0 (0 to 1)		
	CAF + L-PRF	3 (2 to 3)	1 (0 to 1)		
	Between Groups Difference	NS	NS		
**Patient-Related Esthetic Esthetic Score**	CAF + tCTG		9 (8.5 to 10)		
	CAF + L-PRF		9 (9 to 10)		
	Between Groups Difference		NS		
**Overall Treatment Satisfaction**	CAF + tCTG		13 (86%)		
	CAF + L-PRF		14 (93%)		
	Between Groups Difference		NS		

**Table 2:** Maxillary tuberosity—L-PRF differences between estimated mean changes from baseline to 12 months (n = 15 in each group) for the clinical parameters and between differences at follow-up for the remaining ones.

Parameter		Mean Difference ± SE (95% CI)	OR ± Delta SE (95% CI)	*p*-Value
**PD** **(in mm)**		−0.667 ± 0.253 (−1.19 to −0.148)		0.0136
**CAL** **(in mm)**		−0.59 ± 0.29 (−1.19 to 0.00465)		0.0517
**GR** **(in mm)**		0.133 ± 0.171 (−0.218 to 0.484)		0.442
**KT** **(in mm)**		2.2 ± 0.266 (1.65 to 2.75)		<0.001
**GT** **(in mm)**		1.45 ± 0.199 (1.05 to 1.86)		<0.001
**Complete Root Coverage Fraction**		0.133 (−0.21 to 0.48)	1.72 ± 1.32 (0.4 to 7.66)	0.45
**Root Coverage %**	at GR_t0_=2.00	−19.53 ± 10.28 (−40.66 to 1.6)		0.0686
	at GR_t0_=3.47	0.599 ± 5.45 (−10.61 to 11.81)		0.91
	at GR_t0_=5.00	21.55 ± 10.61 (−0.27 to 43.4)		0.0527
**Root Coverage %** **Sensitivity analysis** **(w/o cases 7, 13)**	at any GR_t0_	8.3 ± 3.64 (0.801 to 15.8)		0.0314
**RES score**		−0.207 ± 0.341 (−0.875 to 0.462)		0.54
**Discomfort** **(VAS)**		0.588 ± 0.364 (−0.125 to 1.3)		0.11
**Dentine** **Hypersensitivity**		−0.067 ± 0.182 (−0.424 to 0.29)		0.71
**Patient-related esthetic score (VAS)**		−0.41 ± 0.677 (−1.74 to 0.917)		0.54
**Overall treatment** **satisfaction**		−0.067 (−0.28 to 0.14)	0.464 ± 0.596 (0.037 to 5.75)	0.55

## Data Availability

The data are available upon request from the authors.

## Supplementary Materials

The following supporting information can be downloaded at: https://www.mdpi.com/article/10.3390/dj12040086/s1.
